# Quantifying Next Generation Sequencing Sample Pre-Processing Bias in HIV-1 Complete Genome Sequencing

**DOI:** 10.3390/v8010012

**Published:** 2016-01-07

**Authors:** Bram Vrancken, Nídia Sequeira Trovão, Guy Baele, Eric van Wijngaerden, Anne-Mieke Vandamme, Kristel van Laethem, Philippe Lemey

**Affiliations:** 1 Rega Institute for Medical Research, Clinical and Epidemiological Virology, Department of Microbiology and Immunology, KU Leuven—University of Leuven, 3000 Leuven, Belgium; Nidia.SequeiraTrovao@rega.kuleuven.be (N.S.T.); guy.baele@rega.kuleuven.be (G.B.); annemie.vandamme@uzleuven.be (A.-M.V.); kristel.vanlaethem@uzleuven.be (K.L.); philippe.lemey@rega.kuleuven.be (P.L.); 2 University Hospitals Leuven, 3000 Leuven, Belgium; eric.vanwijngaerden@uzleuven.be; 3 Center for Global Health and Tropical Medicine, Unidade de Microbiologia, Instituto de Higiene e Medicina Tropical, Universidade Nova de Lisboa, 1349-008 Lisbon, Portugal

**Keywords:** HIV, full genome sequencing, NGS

## Abstract

Genetic analyses play a central role in infectious disease research. Massively parallelized “mechanical cloning” and sequencing technologies were quickly adopted by HIV researchers in order to broaden the understanding of the clinical importance of minor drug-resistant variants. These efforts have, however, remained largely limited to small genomic regions. The growing need to monitor multiple genome regions for drug resistance testing, as well as the obvious benefit for studying evolutionary and epidemic processes makes complete genome sequencing an important goal in viral research. In addition, a major drawback for NGS applications to RNA viruses is the need for large quantities of input DNA. Here, we use a generic overlapping amplicon-based near full-genome amplification protocol to compare low-input enzymatic fragmentation (Nextera™) with conventional mechanical shearing for Roche 454 sequencing. We find that the fragmentation method has only a modest impact on the characterization of the population composition and that for reliable results, the variation introduced at all steps of the procedure—from nucleic acid extraction to sequencing—should be taken into account, a finding that is also relevant for NGS technologies that are now more commonly used. Furthermore, by applying our protocol to deep sequence a number of pre-therapy plasma and PBMC samples, we illustrate the potential benefits of a near complete genome sequencing approach in routine genotyping.

## 1. Introduction

Determining the most efficient antiretroviral treatment options through genotypic testing for the presence of resistance-associated mutations (RAMs) has become common practice in the routine management of HIV infection. Studies addressing the sensitivity and accuracy of standard genotypic tests have revealed that RAMs present at levels below 20% are generally not detected and that there is considerable uncertainty about the estimated proportion of detected RAMs that are present in less than 50% of the population [1,2,3,4]. This is a major limitation, because minority drug-resistant subpopulations can limit therapy efficacy [5,6,7,8,9,10,11,12,13]. In addition, the outgrowth of (undetected) minority variants with RAMs under drug selective pressure increases the risk of transmission of drug resistance, which is also associated with therapy failure [14,15].

The integrated state of HIV is an important feature of the viral life cycle in the context of resistance to antiretroviral therapy, because it allows drug-resistant variants to persist as archived provirus in peripheral blood mononuclear cells (PBMCs). Consequently, even though drug resistance can be undetectable in the plasma compartment, this “memory” reservoir can exert a long-term impact on responses to antiretroviral therapy [16,17]. Yet, routine genotyping tests generally focus on currently circulating viral variants by examining the plasma fraction of blood.

Currently-used clinical drug resistance tests also focus on a limited number of small genomic fragments. There is, however, a growing need to simultaneously probe multiple genomic regions due to the development of drugs that target different steps in the viral life cycle [18]. In recognition of this need, studies that combine both the full genome and deep sequencing dimensions have already been reported [19,20,21,22,23,24,25,26]. Yet, the need for relatively large quantities of input DNA represents an important drawback for deep sequencing applications to RNA viruses. To meet these requirements, most researchers initially resorted to a sequence-specific amplification and sequencing of small genomic fragments following RT-PCR amplification of the target(s) of interest [27]. While this method allows the incorporation of platform-specific adaptors and, if required, barcodes for multiplexing during the PCR [28], it may introduce systematic biases, as well as stochastic error [29,30,31,32]. Small amplicon-based approaches cannot readily be extended to full genome sequencing, because targeting numerous overlapping small amplicons becomes impractically labor-intensive and cost prohibitive. This is further aggravated by the need to perform replicate analyses in order to minimize the potential impact of stochastic fluctuations [33,34]. Two alternatives have been suggested to at least partly address these problems. Willerth *et al*. [20] demonstrated that HIV genomes from cell culture supernatant can be amplified and sequenced on the Illumina platform using random-priming strategies, which were adopted from transcriptome analysis. Similar promising approaches directly target the viral RNA with improved versions of the same or other sequence-independent amplification protocols [25,26]. Whereas these approaches should be less prone to amplification-associated biases (but see [35]) and have proven useful, it remains to be established how well these systems perform on (challenging) clinical samples in a direct comparison to highly-optimized RT-PCR based methods, the current gold standard in routine clinical care. An alternative approach is to amplify only a small set of large overlapping amplicons covering the genome [19,21,22,23], albeit with potential biases due to sequence-specific amplification. Both sequence-specific and random amplification methods may generate fragments that are too long for efficient clonal amplification and sequencing. The sample DNA is typically fragmented to the correct size range using mechanical (sonication or nebulization) or enzymatic approaches (e.g., Nextera™ or NEBNext Fragmentase), but all of these suffer from inherent biases [36,37].

Here, we make use of a sequence-specific amplification strategy for in-depth complete genome characterization in clinical HIV-1 samples on the Roche 454 platform. We adopt a strategy similar to Bimber *et al*. [19] and Henn *et al*. [22] and generate six large overlapping amplicons to cover the entire HIV-1 genome, but similar to Gall *et al*. [21], we extend the applicability of this approach by implementing (RT-) PCR protocols optimized for the generic detection of HIV-1 group M (responsible for the worldwide epidemic) [38,39,40,41,42]. In the next step, we compare an enzymatic fragmentation method, the Nextera™ transposon-based technology that has low DNA input requirements, to conventional mechanical Roche 454 shearing. By examining pre-therapy plasma and PBMC samples from patients of a small transmission chain [43] and from a patient who failed first-line therapy because of an undetected low-level drug resistance mutation [5], we demonstrate the utility of our approach in hypothesis-driven research.

## 2. Experimental Section

### 2.1. Samples

We studied the viral population in a small HIV-1 subtype B transmission chain (involving three patients: AR01, AR05 and AR07) [43] and in one additional patient [5] based on three plasma samples, two PBMC pellets and the plasma-derived outer PCR product of amplicons gag-PR, p2-RNAseH, IN-Vif, Env and Nef. An overview of the available samples is presented in Table 1. The patients of the transmission chain were all infected by dual-class resistant HIV-1. Patient AR06 failed first line therapy because of an undetected minority variant. The research was conducted according to the Declaration of Helsinki, and the use of patient samples was approved by the medical ethics committee of the University Hospitals Leuven, Leuven, Belgium (Reference B322201420270). 

**Table 1:** Overview of the available samples.

Patient	Plasma	PBMC Pellet	Outer PCR Product *
AR01	no	yes	yes
AR05	yes	no	yes
AR06	yes	no	no
AR07	yes	yes	yes

* No outer PCR product for amplicon Vif-Vpr-Vpu was available for any of the samples (see also Supplementary Materials, Table S1).

### 2.2. RNA Extraction

A starting volume of 140 μL plasma was eluted in 60 μL of elution buffer from the QIAamp Viral RNA Mini Kit (Qiagen, Venlo, The Netherlands) as described in the manufacturer’s spin protocol. Viral RNA was extracted in six replicates and pooled to minimize sampling effects [33] and stored at −80 °C.

### 2.3. DNA Extraction

DNA was extracted once using the QIAamp Blood DNA Mini Kit (Qiagen) according to the spin protocol. To ensure purification of RNA-free DNA, our protocol included the optional addition of RNase A (Qiagen).

### 2.4. Reverse Transcription and PCR Amplification

The coding part of the HIV-1 genome of the viral and proviral samples was amplified using a set of 6 overlapping amplicons (Supplementary Materials, Figure S1). (RT-) PCR amplifications were also replicated and pooled before proceeding to the next step (Supplementary Materials, Table S1) [34]. The strategy of pooling replicate RNA extractions ensured that we obtained sufficient volume for the 5-replicate cDNA synthesis of all amplicons, except for patient AR01. Due to the absence of stored plasma for this patient, we had to resort to a single plasma-derived outer PCR product (Table 1 and Supplementary Materials, Table S1).

The first round amplification of the PBMC samples was performed with the same master mixes as used for reverse transcription and amplification of plasma samples. The cycling conditions were also identical, except for the omission of the RT step (resulting in a hot-started cycling program). The extraction volume we obtained did not permit 5-replicate cDNA synthesis with the usual 10 μL extract as the input. Instead, to arrive at the same number of replicates, 6 μL of DNA extract were complemented with 4 μL of H${}_{2}$O, except for Nef. Here, we added 10 μL of the DNA extract, because the test with the 6-μL approach yielded no positive result as determined on a 1% agarose gel. Because of this, only 4 replicate outer PCR reactions were possible for Nef.

All reactions were performed in a Biometra T3000 thermal cycler. An overview of all (RT-) PCR mixes, cycling conditions and a list of primers (synthesized by Eurogentec) is provided in the Supplementary Materials, Table S2 and Figures S2 and S3. The quality of the PCR products was visually assessed through gel electrophoresis on a 1% agarose gel.

### 2.5. Purification and Quantification

PCR products from the plasma and PBMC samples were pooled, purified and quantified before further processing using the different fragmentation methods. The sample fraction for fragmentation using the general 454 library preparation was purified with the Illustra GFX PCR DNA and Gel Band Purification kit (GE Healthcare, Diegem, Belgium). The fraction fragmented with the Nextera™ protocol was purified with DNA Clean & Concentrator (Zymo Research, Freiburg, Germany). All quantifications were done with the Quant-iT dsDNA HS Assay Kit or Quant-iT dsDNA BR Assay Kit from Invitrogen (Waltham, MA, USA). Upon quantification, all amplicons from the same sample were pooled in an equimolar fashion.

### 2.6. Fragmentation

Five micrograms of the pooled inner PCR product of both the RNA and DNA samples was subjected to fragmentation by mechanical shearing according to the general 454 library preparation protocol (Roche Diagnostics, Vilvoorde, Belgium) while incorporating multiplex identifier tags (MIDs). The tagmentation reaction with the Nextera™ DNA Sample Prep kit (Roche Titanium compatible, Epicentre Biotechnologies, Madison, WI, USA) was performed according to the manufacturer’s protocol and resulted in a sequencing-ready bar-coded library. The modest input requirement of 50 ng allowed the use of both the outer and inner PCR products of all samples for comparison, except for the plasma sample from patient AR01, because the stored outer PCR product did not contain sufficient DNA.

### 2.7. Sequencing and Data Analysis

Before proceeding with the sequencing, we assessed the quality of the libraries using capillary electrophoresis (Agilent BioAnalyzer, Agilent, Diegem, Belgium). Because the fragment size distribution of the Nextera™ libraries was skewed, we tested two emulsion PCR (emPCR) conditions (0.15 and 0.30 copies per bead (cpb)) on the inner PCR product of the PBMC sample from patient AR01. Due to a technical error, no deep sequencing data could be generated for the Nextera™ fragmented plasma inner PCR product of patient AR07. An overview of the number of reads for each sample is provided in the Supplementary Materials, Table S3.

Sequencing was carried out by Genomics Core (University Hospitals Leuven, Leuven, Belgium) on the GS-FLX 454 pyrosequencing platform (Roche Applied Science, Vilvoorde, Belgium) with Titanium chemistry, and the results were provided as Standard Flowgram Files . Sequence data were extracted and converted to FASTA and QUAL-format with a freely-available Python script [44]. During this format conversion, reads were clipped at the transition of high to low quality base calls at the site recommended by the 454 software. Prior to the read cleaning with RC454 [22], reads with an exact match to both the barcode and, for the Nextera™-fragmented samples, the transposon end sequences were extracted with Segminator II [45] and assigned to the corresponding sample. When available, we used patient-specific reference sequences obtained by Sanger sequencing to map the reads (see Supplementary Materials, Table S4). *De novo* assembled sequences obtained by VICUNA [46] were used to map the reads in the remaining genome regions. We used the the V-Phaser algorithm [22] in an attempt to distinguish sequencing errors from true variation.

## 3. Results

We first report on the degree of variability associated with the emPCR and sequencing steps. Next, we compare the variation between the Nextera™ fragmentation method and conventional mechanical shearing in the sample comparisons. Finally, we construct a near complete genome resistance profile for clinical plasma and PBMC samples.

### 3.1. emPCR/Sequencing Associated Variability

Because of the cautionary approach to test two emPCR conditions and due to low coverages after the first run, a number of Nextera™ fragmented samples were clonally amplified and sequenced in duplicate (Supplementary Materials, Table S3). To score the concordance between the results from these duplicates, we calculated the fraction of positions at which the difference in detected frequency of all nucleotides is at most 1%, 5% and 10% (Table 2). These fractions are on average 87.94%, 98.73% and 99.72%, respectively. To visualize this variation, we plotted the largest difference in observed nucleotide frequency along the axis of the patient-specific reference sequence for these samples (Figures S4–S9). In accordance with the highly similar results, the majority rule consensus sequence differed at only 15 positions in the six samples under comparison (median: three; range: 1–4). In 12/15 (80%), this could be attributed to a nearly 50%-50% mixture of two variants, where a small difference can tip the balance in favor of one nucleotide. We also noted a few outliers where the difference in the detected proportion of a nucleotide amounts to ≥20%, which represent 0.01%–0.07% of all positions in the compared samples. Of these, eight (34.78%) are located within or adjacent (±5 nt) to homopolymers (length ≥4).

**Table 2:** Proportions of sites with nucleotide differences below 1%, 5% and 10% for various sample comparisons.

Patient	Sample	1%	5%	10%
	emPCR/sequencing			
AR01	PBMC inner PCR ${}^{a}$	86.78	99.41	99.92
AR05	plasma outer PCR	92.56	99.30	99.88
AR06	plasma outer PCR	85.69	98.72	99.84
	plasma inner PCR 1a-2a ${}^{b}$	84.03	98.17	99.59
	plasma inner PCR 1b-2b ${}^{b}$	87.89	98.38	99.63
AR07	PBMC outer PCR	88.21	98.61	99.65
	plasma outer PCR	90.48	98.51	99.53
	*average*	*87.94*	*98.73*	*99.72*
	fragmentation method			
AR01	PBMC inner PCR	78.90	96.34	99.29
	plasma inner PCR	90.30	98.68	99.61
AR05	plasma inner PCR	88.90	99.14	99.80
AR06	plasma inner PCR	81.80	97.97	99.49
AR07	PBMC inner PCR	85.66	97.33	99.07
	*average*	*85.11*	*97.89*	*99.45*

Only positions with coverage ≥100 for both samples are taken into account. When possible, replicate sequence data were pooled when comparing the fragmentation methods. ${}^{a}$ Data for this sample were obtained from the same run, but with two emulsion PCR (emPCR) conditions (0.15 and 0.30 copies per bead (cpb)). ${}^{b}$ Due to a technical error, the inner PCR product of the plasma sample of patient AR06 was sequenced twice at both sequencing runs (with different multiplex identifier tags (MIDs)) (see also the Supplementary Materials, Table S3).

This variability likely stems from the random disproportional attachment of templates to empty beads during the emPCR step [47] or from errors arising during the actual sequencing process. Because many of the factors that determine the pyrosequencing error rate (e.g., position in the sequence, size of the template and spatial localization on the picotiter plate (PTP) [48]) vary between sequencing experiments, we considered this source of error as essentially stochastic. For this reason, and similar to the strategy of pooling extracts and (RT-) PCR products, we pooled the sequence data for samples from both runs for the remaining analyses.

### 3.2. Comparison of Nextera™ with Standard Shearing

We compared the compositional differences between both fragmentation methods, Nextera™ and standard shearing, in the same way as above (Table 2). The fraction of sites where the largest difference in the detected percentage of any of the nucleotides amounts to 1%, 5% and 10% is on average 85.11%, 97.89% and 99.45%, respectively. Per category, this is 2.83%, 0.84% and 0.27% less when compared to the mean emPCR/sequencing-associated variability. The consensus sequences (majority rule) of the five Nextera™ and standard shearing fragmented samples differs at 20 positions (median: three; range: 1–9), which can be attributed to a nearly 50%-50% mixture of two nucleotides in 17 cases (85%). Positions with a difference in frequency of a nucleotide ≥20 represent 0.04% to 0.15% of all positions in the compared samples; 10 (50%) of these are associated with homopolymers (length ≥4). A visual representation of the variability of when comparing fragmentation methods is provided in the Supplementary Materials, Figures S10–S14.

### 3.3. Resistance Profiling

We investigated the potential of our near full genome genotyping approach by creating a RAM profile for each patient using the Stanford list with major HIV-1 drug resistance mutations [49], complemented with the list of reverse transcriptase (RT) and protease (PR) mutations used for drug resistance surveillance [50]. While the former list includes RAMs spread over the entire genome, the latter focuses on the protease and RT region and includes additional sentinel mutations. To illustrate that no (low-frequency) RAMs were detected at most positions associated with drug resistance, we provide the complete RAM profiles of the four patients in the Supplementary Materials, Tables S5–S8.

Data obtained by traditional cloning and Sanger sequencing were available for the PR and RT regions for all patients from the same sample that was used for 454 data generation, as was pre-therapy follow-up data for the transmission chain patients (Supplementary Materials, Tables S4–S7) [43]. A comparison highlights that all ubiquitously-present (≥80%) RAMs were detected in similar proportions irrespective of the sequencing and fragmentation technique. When contrasting the cloned and NGS data, most differences remain limited to 1%, and 10/16 (62.5%) were completely fixed according to all approaches. There are two notable outliers: nucleoside reverse transcriptase inhibitor (NRTI) position 41L and non-nucleoside reverse transcriptase inhibitor (NNRTI) position 179D of patient AR06, where the difference amounts to 4% and 10.28%, respectively. Some minority variant RAMs (≤20%) were only detected by the pyrosequencing approach (*n* = 12; median: four; range: 2–6). In contrast, in each patient, one minority variant RAM was only picked up by the cloned sequence data. The resistance profiles obtained by Nextera™ fragmentation and standard shearing also are very similar, and all unique RAM calls again only involve RAMs at levels ≤20%.

The low input requirements of the Nextera™ fragmentation method enabled profiling the outer PCR products of a number of samples (Table 2 and Supplementary Materials, Table S3). When contrasted with the resistance profiles obtained from the matching Nextera™ fragmented inner PCR products, all differences in proportions of RAMs were below 5%.

To compare the diversity of the PBMC and plasma compartments, the amino acid content profiles following Nextera™ and standard shearing fragmentation were combined, and a histogram of the diversity per position was created (Supplementary Materials, Figures S15 and S16). This reveals a number of subpopulations in the PBMC compartment of patient AR01 that were not detected in the plasma sample. The higher diversity of the PBMC reservoir is reflected in a mean of 2.01 different amino acids per position, *vs.* 1.37 in the plasma. For patient AR07, the diversity of the PBMC reservoir is also higher, but less distinct from that of the plasma viruses: the average number of amino acids per position is 1.75 for the PBMC and 1.56 for the plasma reservoir. The resistance profiles for both reservoirs reveal the same pattern for minority variants as observed in the previous comparisons. However, a number of moderately prevalent RAMs (≥20%) are only seen in the PBMC reservoir. Specifically, this concerns NRTI substitution 184I and fusion inhibitor substitution 36S for patient AR01, and for patient AR07, this involves NNRTI substitution 190E.

## 4. Discussion

Massively parallel sequencing methods enable studying the composition of complex viral populations in a cost-effective fashion and point to the potential for near full-length genotyping approaches in routine clinical practice [51,52,53,54]. In order to take up an important role in decision-making in patient care, NGS-based genotyping approaches need to demonstrate excellent sensitivity and accuracy. It is therefore no surprise that much effort has been invested to investigate biases at all stages from sample to sequence data. Here, we compared two fragmentation methods, Nextera™ fragmentation and standard shearing, for a near complete genome characterization based on a new sequence-specific amplification protocol.

In agreement with earlier findings that show similar results for different fragmentation methods [55], we find a similar population composition for both fragmentation procedures. Nevertheless, we recommend the Nextera™ fragmentation, because the lower DNA input requirements allow sequencing the product of a single PCR amplification, which avoids the risk of biases associated with an additional sequence-specific amplification of nested PCR protocols. Interestingly, the DNA input requirement for Nextera™ has further decreased from 50 ng since the start of this study to 1 ng at present, which enables examining more challenging samples. Recently, it also became possible to anticipate elongation and sequencing errors in the experimental sample pre-processing procedures. Some of these procedures rely on attaching unique identifiers to each DNA fragment, either by tagging the primer [56] or sequencing adaptors [57] with a unique barcode. Others procedures rely on the rolling circle amplification of the input DNA or RNA molecules [58,59]. They all result in sequence reads that can be grouped either by the shared unique barcode or by the physical linkage and allow for creating consensus sequences for all reads that represent the same original template. An additional advantage of early-stage tagging [56] over later-stage tagging [57] and rolling circle amplification [58,59] is that the former can also identify template resampling and can be used to reconstruct the original population structure with greater confidence.

Replicate sequencing of some samples reveals that most of the variability results from the emPCR/sequencing steps rather than from the fragmentation methods (Table 2). The introduction of substantial variability after the fragmentation step in the sample pre-processing procedures highlights that in order to arrive at high levels of confidence in the observed frequencies of (minority) variants, it is crucial to average out random errors by pooling replicates at every stage of the pre-processing protocol and not only at the extraction and amplification stages, the importance of which was previously shown [33,34]. Specific attention should be paid to this when accurate frequency estimates of minority variants are required, such as in establishing the clinically-significant cutoff values. Although Roche announced it will phase out the 454-pyrosequencing platform, we believe these results remain relevant for other NGS technologies because of the stochastic nature inherent to the clonal amplification and sequencing steps. As these steps are conceptually the same for the Ion Torrent platform, we specifically anticipate similar issues for this technology. Many more sequence reads per sample can be generated by Illumina platforms, which is currently the most widely-used NGS method. Because this decreases the potential bias of stochastic events, we anticipate that random effects that occur during the bridge amplification and sequencing steps will be less pronounced. The third generation of sequencing technologies (TGS, such as the already available PacBio SMRT and Oxford Nanopore minION systems) aim at directly determining the nucleotide order in the fragments of interest, making the massively parallel clonal amplification step redundant [60]. This considerably simplifies the sample preparation protocols, which reduces to an extraction and amplification step to arrive at sufficient amounts of input material. With an appropriate experimental setup (e.g., pooling of replicate extraction and amplification products [33,34]), the risk of introducing artifacts can be greatly reduced. Nonetheless, it is well acknowledged that there always remains some unexplained variation (the so-called “batch effect” [61,62]), and this may become important when investigating the lower end of the frequency distribution of variants, independent of the NGS technology.

The TGS technologies also offer longer read lengths, which can in theory lead to complete variant sequencing without the need for assembly. However, the reduced sensitivity of long-range PCRs and the current error rates (∼4% for minION and ∼11% for single pass long reads in SMRT sequencing) currently prevent a sensitive screening of complex virus populations. On the other hand, the excellent base calling accuracy that can be achieved with the SMRT circular consensus sequencing method, at the expense of read length, calls for a coupling with sequence-specific and random priming strategies. The current throughput on the PacBio Sequel systems may however be too low to obtain sufficient coverage to compensate for the high levels of host contamination in the samples with the latter approach.

The relatively modest impact of the fragmentation and subsequent steps in the sample preparation protocol propagate into the RAM profiles. However, while the variability remains relatively modest for the majority variants, the difference in detected proportion can rise to several fold changes for minority variants. It can therefore be anticipated that the variance in observed proportions of majority variant RAMs introduced at the post-amplification stage will only rarely lead to a qualitative difference in resistance profile interpretation. In contrast, this source of variation will likely become important when determining the clinically-significant cutoffs.

The close agreement in estimated RAM frequencies when comparing matching outer and inner Nextera™ fragmented PCR products (differences are smaller than 5%) may be a consequence of the pooling strategy that levels out the effect of random errors. It is interesting to note that the 184I minority variant RAM reported by Van Laethem *et al.* [5] for patient AR06 was observed in the outer PCR product at the same level (2%), yet disappeared in the inner PCR product. On the other hand, the 184V variant that was linked to therapy failure and detected at 2% [5] could not be identified based on the outer nor inner PCR product. This highlights that caution is needed when scoring the presence/absence of variants at levels close to the detection limit. In addition to experimental variation (see below), a plausible cause for such discrepancies lies in the assumption of uniform error rates for homo- and hetero-polymer regions by the error correction algorithm we used [22], which undoubtedly accounts for a large fraction of the false negative and false positive minority variants.

The fact that the transmission chain patients are still successful on their first-line therapy, whereas patient AR06 experienced therapy failure due to undetected minority variant RAMs seems to add to the conflicting reports on the clinical relevance of minority resistance variants (e.g., [5,6,7,8,9,10,11,12,13,63,64,65]). However, the view has recently emerged that their effect is determined by an interplay between the particular resistance mutations and the specific regimen (low *vs.* high genetic barrier to resistance). In particular, because a single mutation can already lead to high levels of resistance against NNRTIs [66], minority NNRTI RAMs are associated with an increased risk of first-line therapy failure [13,14,67,68,69]. In line with this, patient AR06 started on a therapy with a low genetic barrier to resistance [5], in contrast to the NRTI + protease inhibitor (PI)/ritonavir based first-line regimen of the transmission chain patients. Of note, a thorough characterization of the virus population upon therapy failure may lead to the discovery of additional resistance mutations and can therefore offer potentially actionable information [70,71,72,73]. Because of differences in extraction volume for traditional cloning and sequencing (1 mL) and our NGS protocol (6 × 140 μL), we cannot directly compare the RAM profiles obtained by the different procedures. In particular, we cannot exclude that the minority variant RAMs detected exclusively by the traditional cloning approach are false negatives in the deep sequencing approach.

Our deep sequencing results confirm the earlier finding that all three patients from the small transmission chain (AR01, AR05 and AR07) share dominant (*i.e.*, 100%) dual-class RAMs [43]. Because it is known that drug-resistant variants tend to revert to wild-type upon transmission, we also investigated the available PBMC populations of patients AR01 and AR07 in an attempt to establish which viral reservoir may be best suited to detect (transmitted) drug resistance (TDR) at this stage. The detection of additional (N)NRTI mutations at levels above 20% (184I, patient AR01 and 190E, patient AR07) and a fusion inhibitor mutation (36S, patient AR01) unique to the PBMC compartment highlights the potential advantage of PBMCs for genotyping and also illustrates the potential benefit of a whole genome approach to genotyping. The long-term pre-therapy follow-up data show that none of the above three RAMs and none of the minority variant RAMs from either reservoir evolved to replace the wild-type variants. Of note, four out of five majority variant RAMs persisted in the almost six-year pre-therapy follow-up period for patient AR01, as was first noted by Van Laethem *et al*. [43]. The PBMC compartment also contained a more diverse population in patient AR01 and, to a lesser extent, in patient AR07 than the corresponding plasma compartment. Such differences between patients may reflect a difference between time of infection and time of sampling. In addition, both PBMC populations showed signs of APOBEC3-induced hypermutation as evidenced by multiple mutations of tryptophan to stop codons. Of note, the 184I, 190E and 36S substitutions we reported all involve a G to A transition and are therefore perhaps APOBEC3-induced rather than transmitted RAMs.

The current interpretation of genotypic resistance testing in routine clinical care, which only takes into account the presence/absence of RAMs, is highly efficient in selecting optimal drug combinations. Several lines of evidence, however, suggest that the selection process can be further optimized by including information on the broader genetic background. For example, Boltz *et al.* [63] show that low-frequency nevirapine (NVP, a NNRTI) RAMs that were selected for under single dose NVP drug selective pressure increase the risk of failing a subsequent NVP-based therapy, which was not observed for the same RAMs that did not emerge under NVP selection pressure. This can be explained by RAMs emerging upon single dose NVP exposure that are linked on the same viral genome. Similarly, coevolution of protease and its substrate Gag during protease inhibitor (PI) exposure can affect PI-based therapy [74,75]. Haplotype reconstruction of NGS data may help to address this, but this remains challenging for short read data [76]. An interesting avenue for further research may be the use of very long-read NGS technologies, such as the PacBio SMRT platform, to reconstruct the haplotypes (e.g., [77]), provided appropriate measures are taken to avoid both PCR-induced [78,79] and *in silico* recombination [76].

In summary, we find that variation introduced during the fragmentation and later steps of the sample preparation can impact the recovery of variant frequencies and that this mostly affects the prevalence estimates of minority variants. Furthermore, our generic near complete genome amplification approach may prove useful in large-scale phylodynamics studies and, after appropriate validation, perhaps also in further studies of the potential benefits of NGS in clinical decision making.

## Supplementary Files

Supplementary File 1
