# Supplementary material for: Quantifying Next Generation Sequencing Sample Pre-Processing Bias in HIV-1 Complete Genome Sequencing
Source: Viruses. 2016 Jan 7;8(1):12. doi: 10.3390/v8010012 (PMC4728572; doi:10.3390/v8010012)
Supplement: Supplementary File 1 [file viruses-08-00012-s001.zip › viruses-102759-supplementary (final)/viruses-102759-supplementary (final).pdf]

Supplementary Materials: Quantifying Next Generation Sequencing Sample Pre-Processing Bias in HIV-1 Complete Genome Sequencing

Bram Vrancken, Nídia Sequeira Trovão, Guy Baele, Eric van Wijngaerden, Anne-Mieke Vandamme, Kristel van Laethem and Philippe Lemey

Table S1. Overview of the amplicons and the number of replicate (RT-)PCR reactions per patient and per sample type.

patient	amplicon name:	gag-PR	p2-RNAseH	IN-Vif	Vif-Vpr-Vpu	Env	Nef
	outer PCR[a]	604 – 2796	1838 – 4650	3881 – 5955	4969 – 6571	5983 – 9144	8356 – 9598
	inner PCR[a]	650 – 2596	1848 – 4637	4189 – 5773	5059 – 6440	6225 – 9057	8514 – 9598
	size [d]	1946	2789	1584	1381	2832	1084
AR01	plasma[b]	[1-0],5	[1-0],5	[1-0],5	-	[1-0],5	[1-0],5
	PBMC[c]	5,5	5,5	5,5	5,5	5,5	4,5
AR05	plasma[b]	[1-5],5	[1-5],5	[1-5],5	[0-5],5	[1-5],5	[1-5],5
	PBMC[c]	-	-	-	-	-	-
AR06	plasma[b]	[0-5],5	[0-5],5	[0-5],5	[0-5],5	[0-5],5	[0-5],5
	PBMC[c]	-	-	-	-	-	-
AR07	plasma[b,e]	[1-5]	[1-5]	[1-5]	[0-5]	[1-5]	[1-5]
	PBMC[c]	5,5	5,5	5,5	5,5	5,5	4,5

[a] The ranges indicate the covered region relative to the HXB2 reference genome.
[b] The numbers between brackets represent the number of respectively old and new RT-PCR reactions that were pooled. The last digit indicates the number of pooled replicate inner PCR reactions.
[c] The number of pooled replicate outer PCR respectively inner PCR reactions.
[d]amplicon length is expressed in nt.
[e]Unfortunately, due to a technical error, no inner PCR product of this patient's plasma sample could be sequenced.

Table S2. Overview of the used primers.

Amplicon		Primer Code	Position in the Genome [a]		Sequence (5'-3')
gag-pr [1]	Outer primers	KVL064	570	603	GTT GTG TGA CTC TGG TAA CTA GAG ATC CCT CAG A
		KVL065	2797	2828	TCC TAA TTG AAC YTC CCA RAA RTC YTG AGT TC
	Inner primers	KVL066	626	649	TCT CTA GCA GTG GCG CCC GAA CAG
		KVL067	2597	2623	GGC CAT TGT TTA ACY TTT GGD CCA TCC
p2-RnaseH [2]	Outer primers	AV190-1	1810	1837	GCT ACA YTA GAA GAA ATG ATG ACA GCA T
		CR1	4651	4687	GAT TCT ACT ACT CCT TGA CTT TGG GGA TTG TAG GGA A
	Inner primers	AV190-2	1817	1847	TAG AAG AAA TGA TGA CAG CAT GYC AGG GAG T
		CR2	4669	4638	CTT TGG GGA TTG TAG GGA ATN CCA AAT TCC TG
in-vif [3]	Outer primers	KVL068	3854	3880	AGG AGC AGA AAC TTW CTA TGT AGA TGG
		KVL069	5956	5981	TTC TTC CTG CCA TAG GAR ATG CCT AAG
	Inner primers	KVL071	5774	5800	CAG AAT TGG GTG YCR ACA TAG CAG AAT
		KVL076	4161	4188	GCA CAY AAA GGR ATT GGA GGA AAT GAA C

Table S2. *Cont.*

Amplicon		Primer Code	Position in the Genome [a]		Sequence (5'-3')
vif-vpr-vpu [b]	Outer primers	KVL144	4943	4968	AGC MAA RCT WCT CTG GAA AGG TGA AG
		KVL145	6572	6603	GTA ACR CAG AGW GGG GTY AAY TTT ACA CAT GG
	Inner primers	KVL146	5030	5058	CAT TAR GGA YTA TGG AAA ACA GAT GGC AG
		KVL147	6441	6466	TTG TGG GTT GGG GTC TGT RGG TAC AC
env [4]	Outer primers	EnvA	5954	5982	GGC TTA GGC ATC TCC TAT GGC AGG AAG AA
		KVL008 *	5284	5308	GGT CAK GGR GTC TCC ATA GAA TGG A
		KVL009	9145	9170	GCC AAT CAG GGA AGW AGC CTT GTG T
	Inner primers	envB	6198	6224	AGA AAG AGC AGA AGA CAG TGG CAA TGA
		envM	9058	9086	TAG CCC TTC CAG TCC CCC CTT TTC TTT TA
nef [b]	Outer primers	KVL072	8330	8355	AAT AGA GTT AGG MAG GGA TAC TCA CC
		KVL073	9599	9620	ACT CAA GGC AAG CTT TAT TGA G
	Inner primers	KVL074	8496	8513	GGA RCC TGT GCC TCT TCA
		KVL073	9599	9620	ACT CAA GGC AAG CTT TAT TGA G

[a] position is relative to the HXB2 reference genome. [b] developed in-house. * PBMC outer sense primer.

Table S3. Impact of MID filtering and RC454 data cleaning.

**standard shearing**		initial # reads	# reads after MID and transposon end sequence filtering	# reads after RC454 cleaning
AR01	PBMC_innerPCR	37,001	36,352 (98.26)	34,640 (93.62)
	plasma_innerPCR	42,055	41,379 (98.39)	40,678 (96.73)
AR05	plasma_innerPCR	21,978	21,410 (97.42)	21,016 (95.62)
AR06	plasma_innerPCR	15,883	15,432 (97.16)	14,856 (93.53)
AR07	PBMC_innerPCR	48,882	47,882 (97.95)	44,334 (90.70)
	plasma_innerPCR	43,661	42,793 (98.42)	41,681 (95.47)
**Nextera fragmentation**				
AR01[a]	PBMC_innerPCR_1	31,787	30,836 (97.00)	28,822 (90.76)
	PBMC_innerPCR_2	33,609	32,555 (96.86)	30,262 (90.04)
	plasma_innerPCR	26,329	25,397 (96.46)	24,366 (92.54)
AR05[b]	plasma_innerPCR	30,124	28,889 (95.90)	27,903 (92.62)
	plasma_outerPCR_r1	20,650	19,051 (92.26)	17,980 (87.07)
	plasma_outerPCR_r2	63,986	57,782 (90.30)	46,925 (74.90)
AR06[b]	plasma_innerPCR_r1a	25,740	24,771 (96.29)	22,809 (88.61)
	plasma_innerPCR_r1b	16,056	14,798 (92.16)	14,077 (87.67)
	plasma_innerPCR_r2a	83,996	78,903 (93.94)	64,108 (76.32)
	plasma_innerPCR_r2b	53,894	50,771 (94.21)	41.322 (76.54)
	plasma_outerPCR_r1	35,047	32,878 (93.81)	27,129 (77.41)
	plasma_outerPCR_r2	106,681	94,066 (88.18)	67,767 (63.52)
AR07[b]	PBMC_innerPCR	27,300	25,257 (92.52)	22,169 (81.21)
	PBMC_outerPCR_r1	34,841	32,995 (94.70)	20,043 (57.53)
	PBMC_outerPCR_r2	122,113	114,212 (93.53)	60,363 (49.43)
	plasma_outerPCR_r1	14,757	13,861 (93.93)	13,017 (88.21)
	plasma_outerPCR_r2	43,185	37,750 (87.41)	31,050 (71.90)

The number of reads per sample before and after the main read cleaning steps is given. The number between brackets corresponds to the fraction of the initial total number of reads that are considered for further analysis.

[a] Data for the PBMC inner PCR product were obtained using two emPCR conditions (0.15 and 0.30 cpb) at the same run.

[b] Data for some of these samples were obtained over 2 runs because the coverage profiles obtained after the first one were indicative for an insufficient in-depth view at multiple positions. The data marked 'r1' were obtained from $1/4^{th}$ PTP during the first run. The data marked 'r2' were obtained from $1/2^{nd}$ PTP during the second run. Due to a technical error the Nextera^{TM} fragmented inner PCR product of sample AR06 was sequenced twice during each run; the subsamples are indicated with "a" and "b".

Table S4. Overview of the available clonal and population sequences.

patient	gag-PR	p2RNAseH	IN-Vif	Vif-Vpr-Vpu	gp160	Nef
AR01	pop	clonal	pop	-	pop	pop
AR05	pop	clonal	pop	-	pop	pop
AR06	-	clonal	-	-	-	-
AR07	pop	clonal	pop	-	pop	pop

pop = population sequence available
clonal = clonal sequence available.
- = no patient-specific sequence available

Table S9. Expected number of difference RNA copies available for cDNA synthesis in relation to the viral load.

patient	viral load[a]	expected input copy number for extraction[b]	expected input copy number for cDNA synthesis[c]	cutoff (%)[d]
		plasma		
AR01	4,876	683	102	0.98
AR05	159,697	22,358	3,726	0.03
AR06	83,176	11,644	1,746	0.06
AR07	57,544	8,056	1,343	0.07
		PBMC		
AR01	10-1,000	9- 860	1-103	100 - 0.97
AR07	30-3,000	18-2,580	3-309	33-0.32

[a]: expressed in copies/mL for the plasma samples. For the PBMC samples, we based the calculations on a proviral load of 1 per 10^3 to 1 per 10^5 PBMCs [5]. For sample AR01 we started from $1*10^6$ and for sample AR07 from $3*10^6$ PBMCs.
[b]: for an extraction starting with 140μL plasma and 200μL PBMC solution.
[c]:based on the minimal advertised recovery yield of 90% at any viral load for plasma samples and 86% for provirus extraction for the used elution volume of 50μL (http://www.qiagen.com/), and assuming 10μL input for the amplification of the plasma samples, and 6μL for the PBMC samples.
[d] the biologically meaningful lower limit of detection.

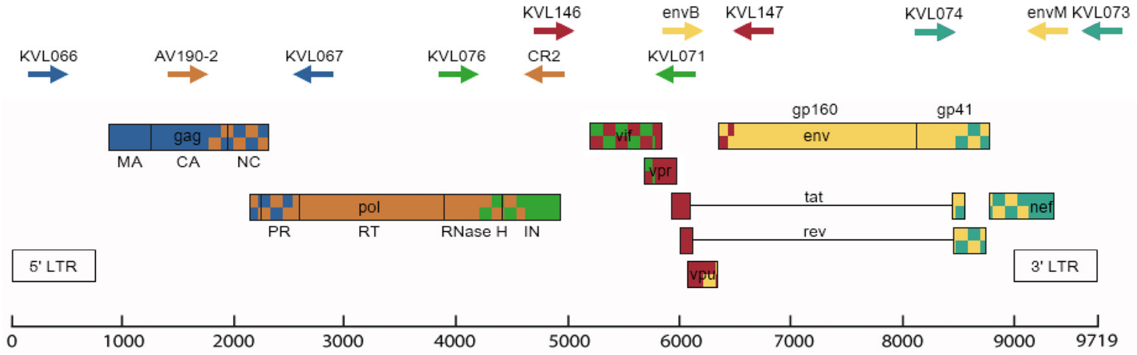

Figure S1. Schematic overview of the overlapping amplicons used for the near full genome amplification of HIV-1. Colored arrows indicate forward and reverse primers, with names corresponding to the primer's names in Table S2.

	Gag-PR			P2-RNaseH			In-Vif		
	concentration \ volume	cycling conditions		concentration \ volume	cycling conditions		concentration \ volume	cycling conditions	
		RNA incubation			*RNA incubation*			*RNA incubation*	
reaction mix	1x	65°C 30''		1x	65°C 30''		1x	65°C 30''	
sense primer	0,2µM	55°C 5'		0,2µM	55°C 5'		0,2µM	55°C 5'	
antisense primer	0,2µM	*Reverse Transcription*		0,2µM	*Reverse Transcription*		0,2µM	*Reverse Transcription*	
$MgSO_4$	0,8mM	55°C 30'		1,3mM	55°C 30'		1,05mM	55°C 30'	
Superscript III/Plat HF	1µL	*PCR cycling profile*		1µL	*PCR cycling profile*		1µL	*PCR cycling profile*	
RNA protector	10U	94°C 2'		10U	94°C 2'		10U	94°C 2'	
RNA/DNA extract	10µL/6µL	94°C 15''	40x	10µL/6µL	94°C 15''	40x	10µL/6µL	94°C 15''	40x
H_2O	add until final	57°C 30'		add until final	61°C 30'		add until final	53°C 30'	
	volume of	68°C 2'		volume of	68°C 3'		volume of	68°C 2'30''	
	50µL	4°C infinite		50µL	4°C infinite		50µL	4°C infinite	

	Vif-Vpr-Vpu			Env			Nef		
	concentration \ volume	cycling conditions		concentration \ volume	cycling conditions		concentration \ volume	cycling conditions	
		RNA incubation			*RNA incubation*			*RNA incubation*	
reaction mix	1x	65°C 30''		1x	65°C 30''		1x	65°C 30''	
sense primer	0,2µM	55°C 5'		0,2µM	55°C 5'		0,2µM	55°C 5'	
antisense primer	0,2µM	*Reverse Transcription*		0,2µM	*Reverse Transcription*		0,2µM	*Reverse Transcription*	
$MgSO_4$	1mM	55°C 30'		/	55°C 30'		1,05mM	55°C 30'	
Superscript III/Plat HF	1µL	*PCR cycling profile*		1µL	*PCR cycling profile*		1µL	*PCR cycling profile*	
RNA protector	10U	94°C 2'		10U	94°C 1'30''		10U	94°C 2'	
RNA/DNA extract	10µL/6µL	94°C 15''	40x	10µL/6µL	94°C 15''	40x	10µL/10µL	94°C 15''	40x
H_2O	add until final	54°C 30'		add until final	55°C 30'		add until final	56°C 30'	
	volume of	68°C 2'		volume of	68°C 2'		volume of	68°C 2'	
	50µL	4°C infinite		50µL	4°C infinite		50µL	4°C infinite	

Figure S2. Overview of the reaction mixes and cycling conditions for the (RT-)PCR reactions.

	Gag-PR			P2-RNaseH			IN-Vif		
	concentration / volume	cycling conditions		concentration / volume	cycling conditions		concentration / volume	cycling conditions	
**mix 1**									
dNTP	200µM			200µM			200µM		
sense primer	0,4µM	*PCR cycling profile*		0,5µM	*PCR cycling profile*		0,4µM	*PCR cycling profile*	
antisense primer	0,4µM	95°C 2'		0,5µM	94°C 2'		0,4µM	95°C 2'	
outer PCR product	5µL	95°C 15''	10x	2µL	94°C 15''	30x	5µL	95°C 15''	10x
		58°C 30'			59°C 30'			55°C 30'	
**mix 2**		68°C 2'30''			68°C 3'			68°C 2'30''	
buffer	1x	95°C 15''	30x	1x	4°C infinite		1x	95°C 15''	30x
$MgCl_2$	2mM	58°C 30''		2mM			2mM	55°C 30''	
Expand HF Enzyme	2,65U	68°C 2'30'' + 5''/cycle		2,65U			2,65U	68°C 2'30'' + 5''/cycle	
	add until final	4°C infinite		add until final			add until final	4°C infinite	
H_2O	volume of			volume of			volume of		
	25µL			25µL			25µL		

	Vif-Vpr-Vpu			Nef		
	concentration / volume	cycling conditions		concentration / volume	cycling conditions	
**mix 1**						
dNTP	400µM			400µM		
sense primer	0,8µM	*PCR cycling profile*		0,4µM	*PCR cycling profile*	
antisense primer	0,8µM	94°C 2'		0,4µM	95°C 2'	
outer PCR product	5µL	94°C 15''	40x	5µL	95°C 15''	10x
		56°C 30'			53°C 30'	
**mix 2**		68°C 1'30''			72°C 1'	
buffer	1x	4°C infinite		1x	95°C 15''	30x
$MgCl_2$	2mM			2mM	58°C 30''	
Expand HF Enzyme	2,65U			2,65U	72°C 1' + 1''/cycle	
	add until final			add until final	4°C infinite	
H_2O	volume of			volume of		
	25µL			25µL		

	Env		
	concentration / volume	cycling conditions	
		PCR cycling profile	
buffer	1x	94°C 1'30''	
sense primer	0,2µM	94°C 15''	5x
antisense primer	0,2µM	55°C 30'	
$MgSO_4$	2mM	68°C 2'45''	
dNTP	200µM	94°C 15''	30x
Plat Taq HF	1U	55°C 30''	
outer PCR product	1µL	68°C 3' + 1''/cycle	
	add until final	4°C infinite	
H_2O	volume of		
	50µL		

Figure S3. Overview of the reaction mixes and cycling conditions for the second round of amplification.

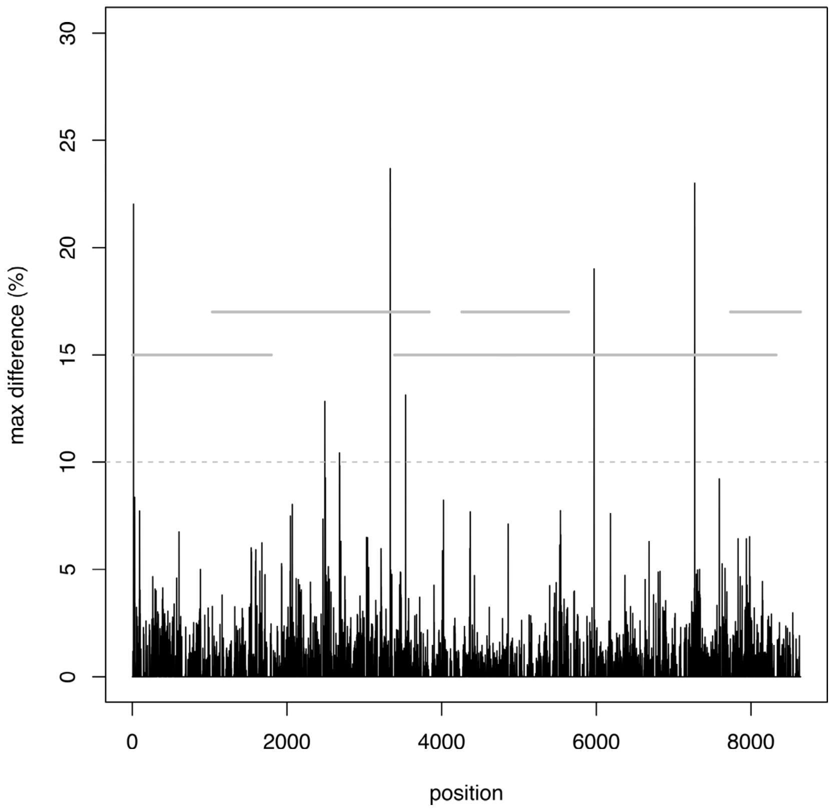

Figure S4. emPCR/sequencing variability of the PBMC inner PCR product for patient AR01. Bars indicate the largest difference in observed nucleotide frequency along the axis of the patient-specific reference sequence. Only positions with coverage ≥10 in both samples are taken into account.

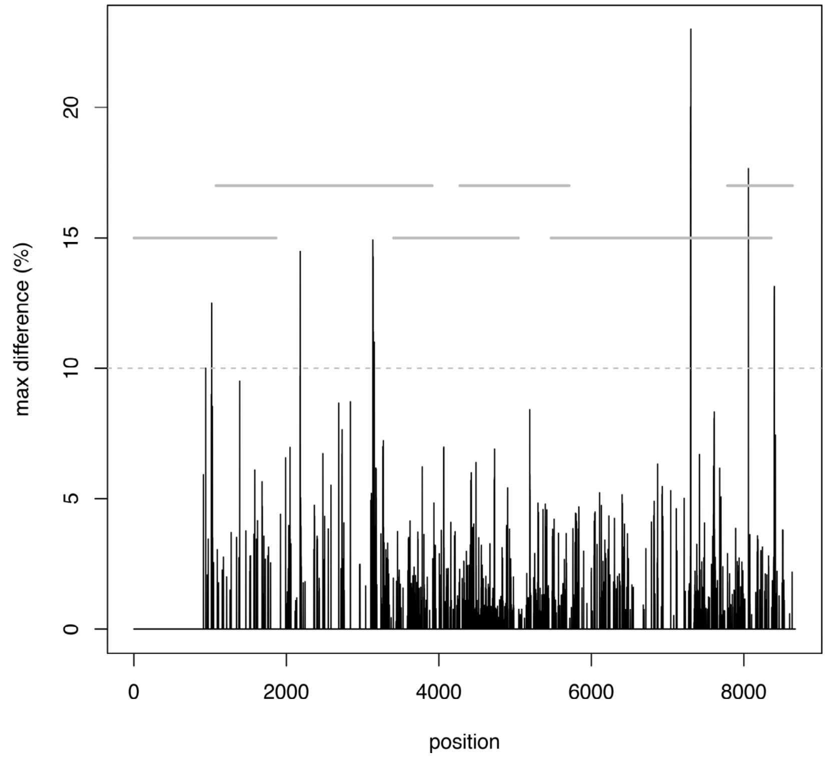

Figure S5. emPCR/sequencing variability of the plasma outer PCR product for patient AR05. Bars indicate the largest difference in observed nucleotide frequency along the axis of the patient-specific reference sequence. Only positions with coverage ≥10 in both samples are taken into account.

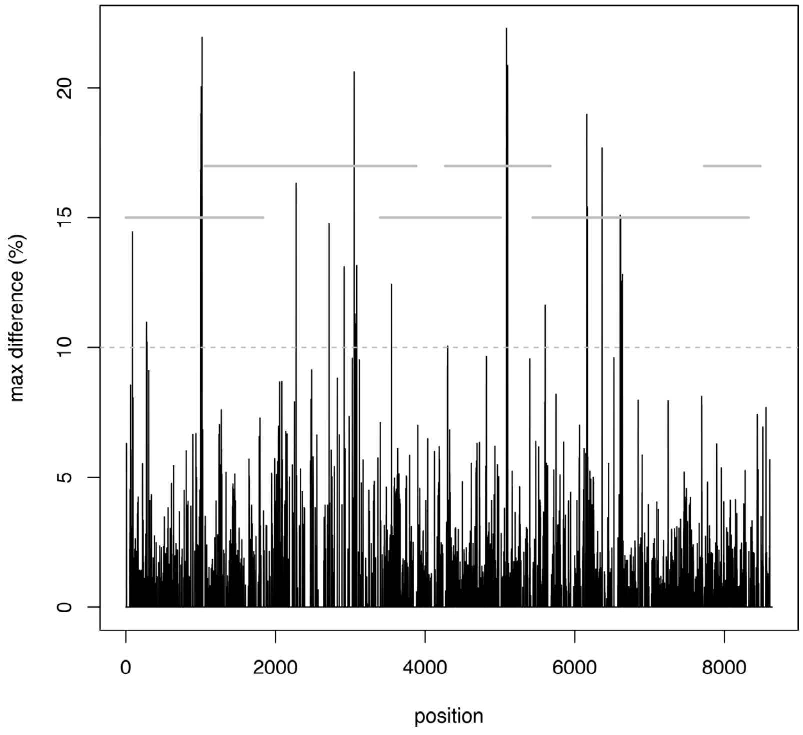

Figure S6. emPCR/sequencing variability of the plasma inner PCR product (1a *vs*. 2a) for patient AR06. Bars indicate the largest difference in observed nucleotide frequency along the axis of the patient-specific reference sequence. Only positions with coverage ≥10 in both samples are taken into account.

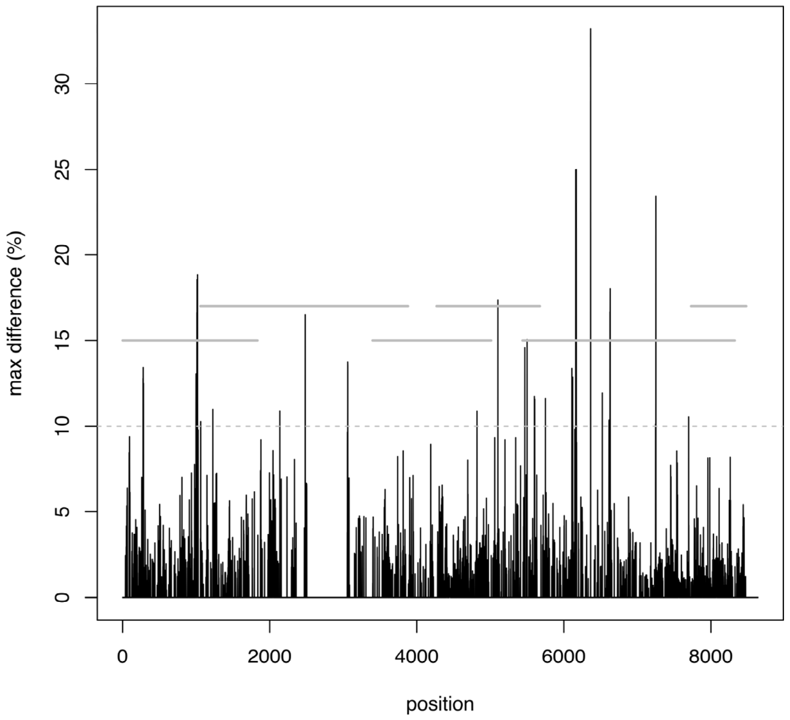

Figure S7. emPCR/sequencing variability of the plasma inner PCR product (1b *vs*. 2b) for patient AR06. Bars indicate the largest difference in observed nucleotide frequency along the axis of the patient-specific reference sequence. Only positions with coverage ≥10 in both samples are taken into account.

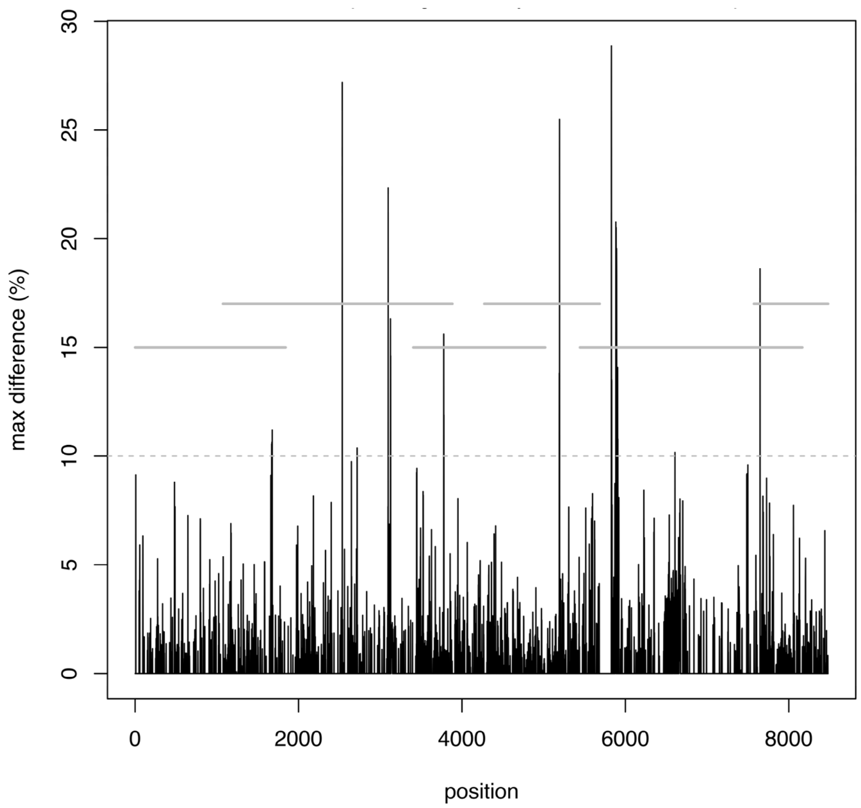

Figure S8. emPCR/sequencing variability of the PBMC outer PCR product for patient AR07. Bars indicate the largest difference in observed nucleotide frequency along the axis of the patient-specific reference sequence. Only positions with coverage ≥10 in both samples are taken into account.

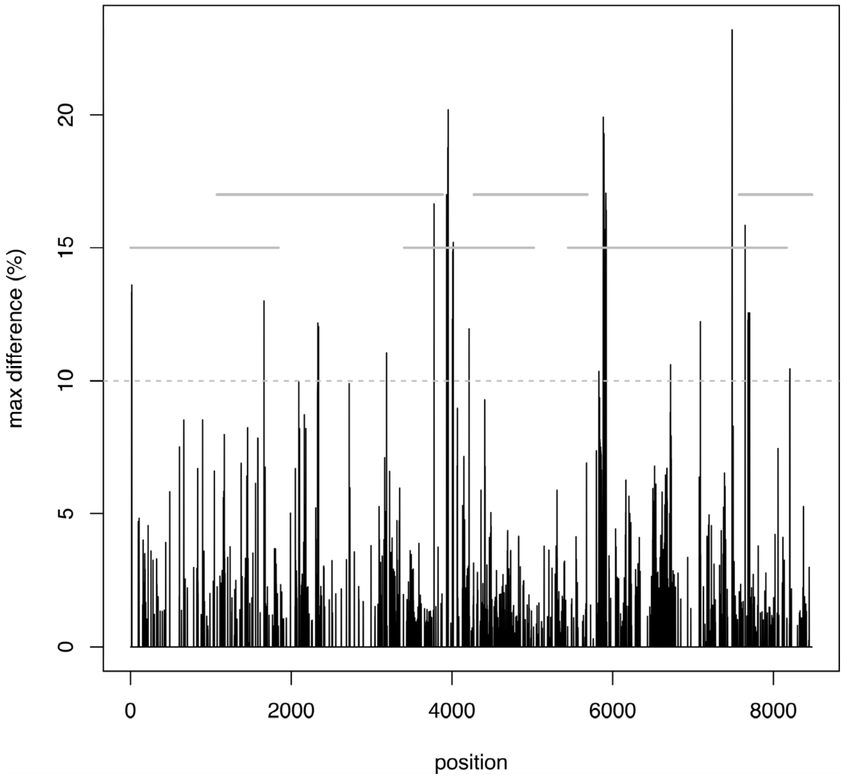

Figure S9. emPCR/sequencing variability of the plasma outer PCR product for patient AR07. Bars indicate the largest difference in observed nucleotide frequency along the axis of the patient-specific reference sequence. Only positions with coverage ≥10 in both samples are taken into account.

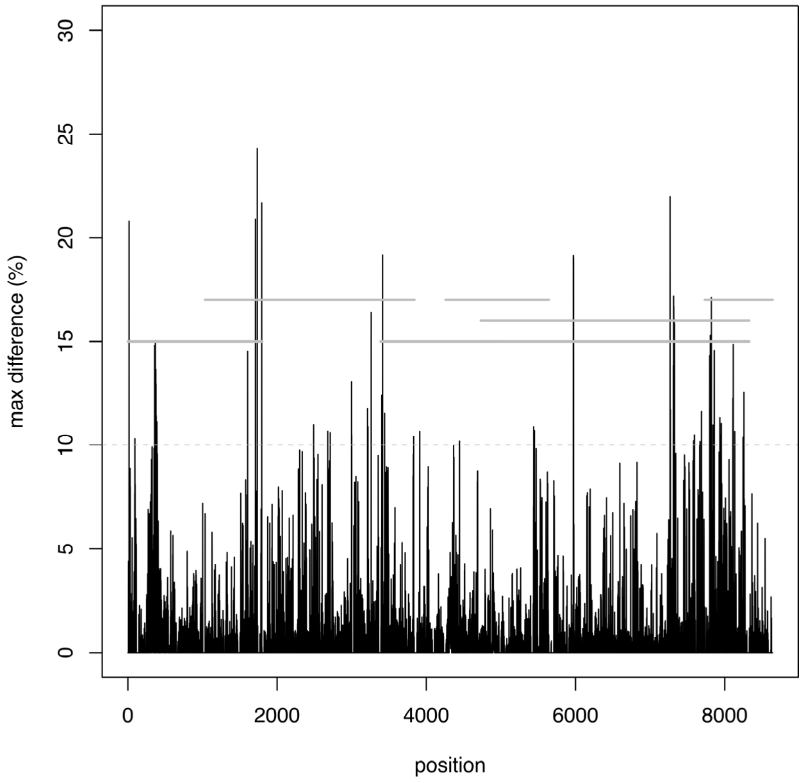

Figure S10. Fragmentation protocol associated variability of the PBMC inner PCR product for patient AR01. Bars indicate the largest difference in observed nucleotide frequency along the axis of the patient-specific reference sequence. Only positions with coverage ≥10 in both samples are taken into account.

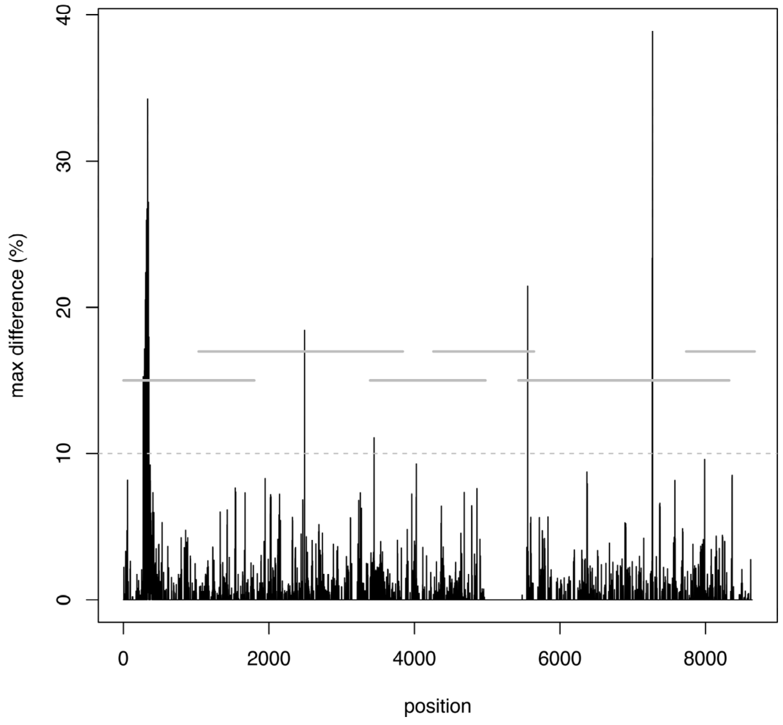

Figure S11. Fragmentation protocol associated variability of the plasma inner PCR product for patient AR01. Bars indicate the largest difference in observed nucleotide frequency along the axis of the patient-specific reference sequence. Only positions with coverage ≥10 in both samples are taken into account.

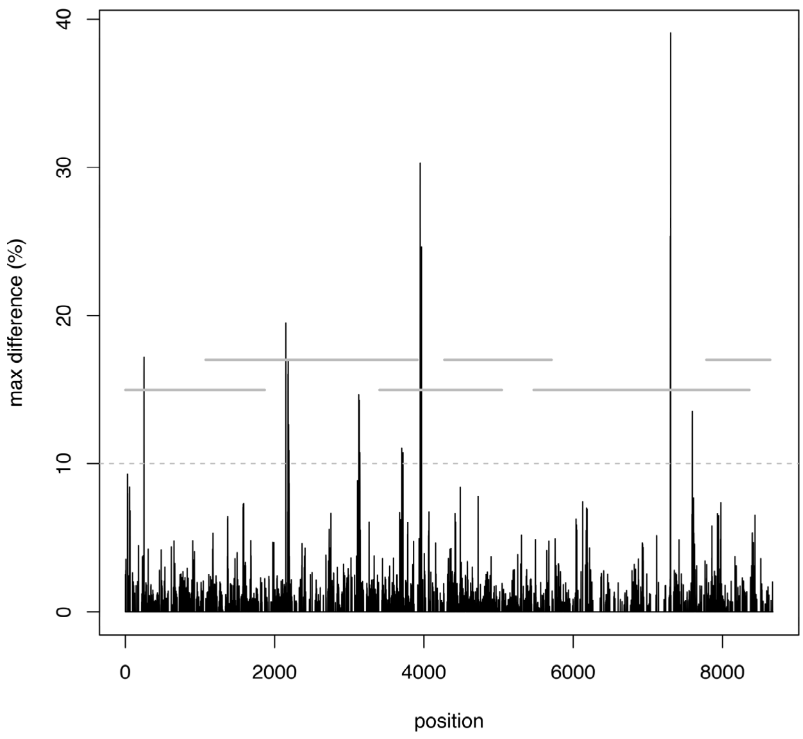

Figure S12. Fragmentation protocol associated variability of the plasma inner PCR product for patient AR05. Bars indicate the largest difference in observed nucleotide frequency along the axis of the patient-specific reference sequence. Only positions with coverage ≥10 in both samples are taken into account.

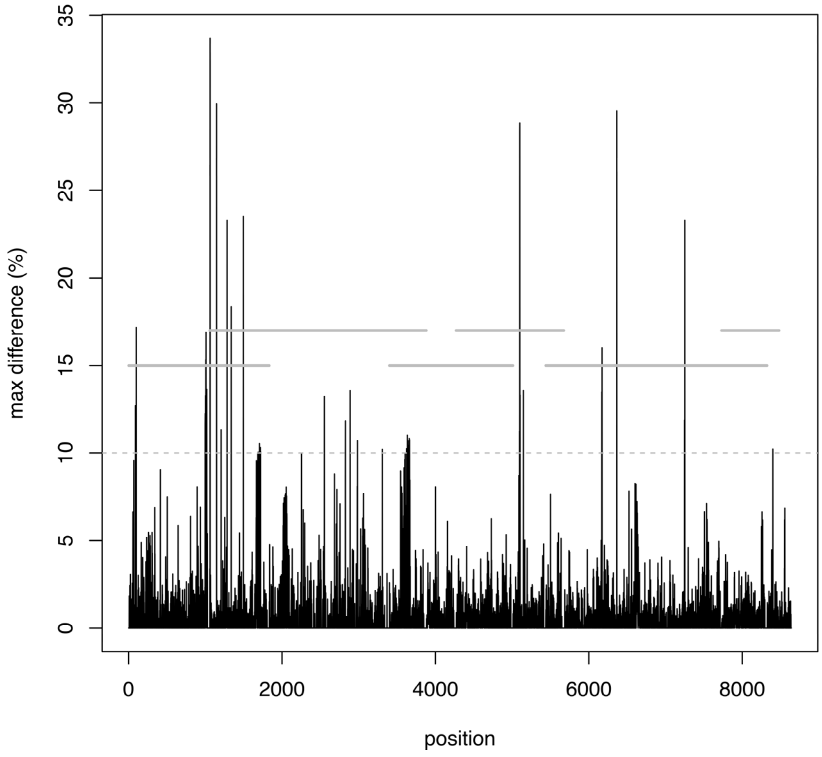

Figure S13. Fragmentation protocol associated variability of the plasma inner PCR product for patient AR06. Bars indicate the largest difference in observed nucleotide frequency along the axis of the patient-specific reference sequence. Only positions with coverage ≥10 in both samples are taken into account.

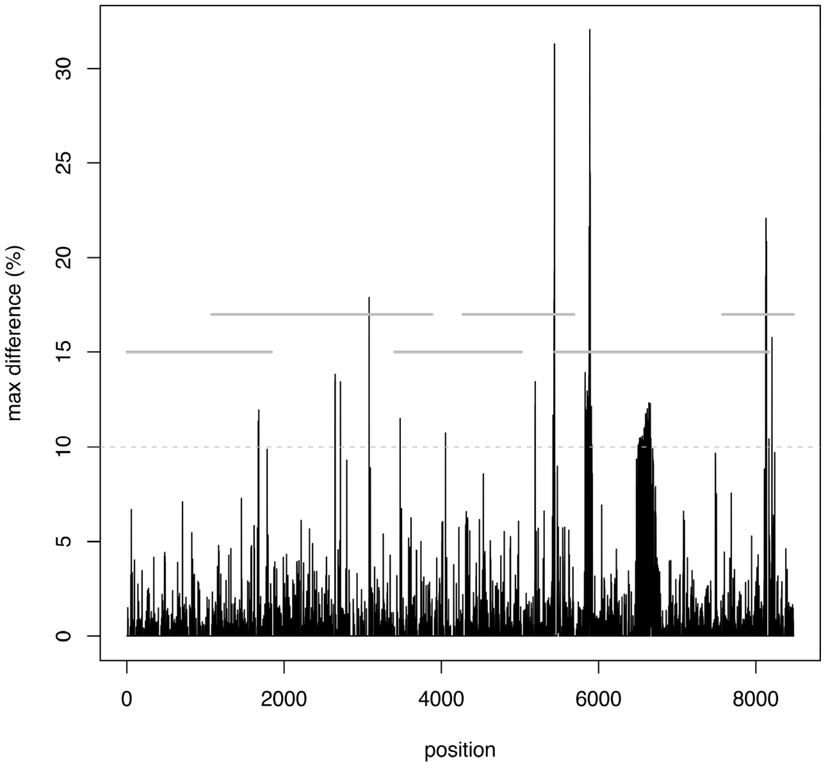

Figure S14. Fragmentation protocol associated variability of the PBMC inner PCR product for patient AR07. Bars indicate the largest difference in observed nucleotide frequency along the axis of the patient-specific reference sequence. Only positions with coverage ≥10 in both samples are taken into account.

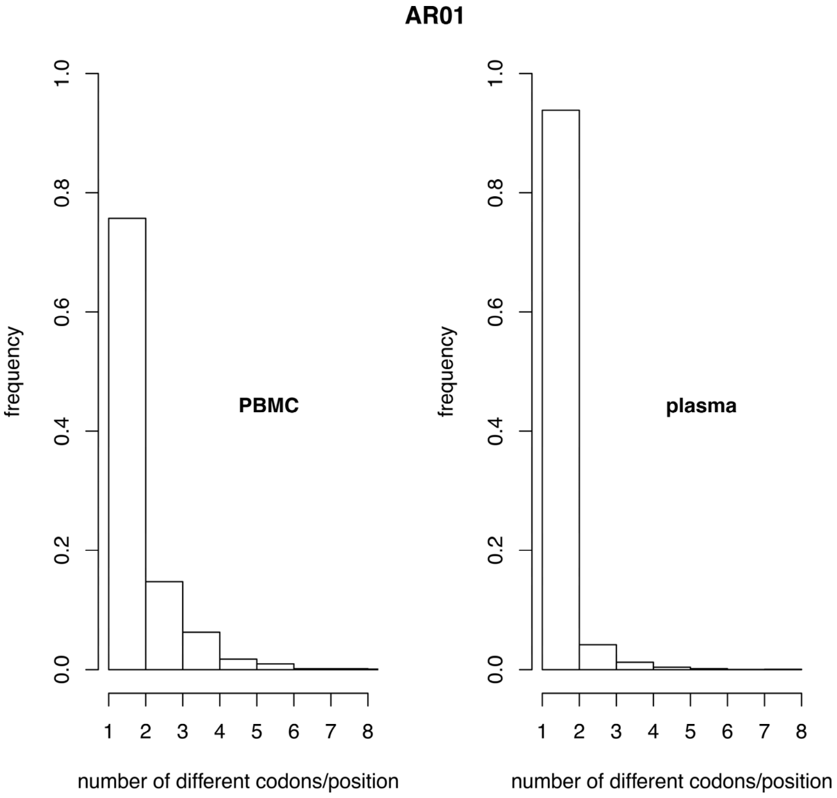

Figure S15. Histograms representing the per position diversity in the PBMC and plasma compartments of patient AR01.

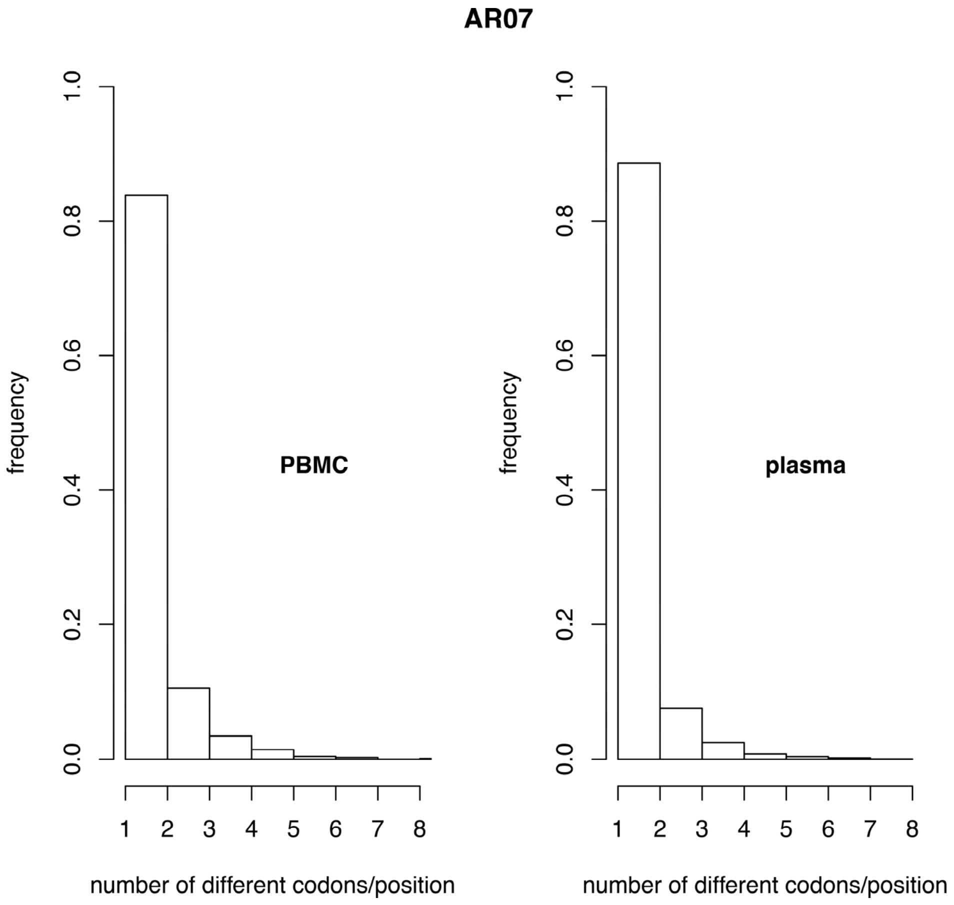

Figure S16. Histograms representing the per position diversity in the PBMC and plasma compartments of patient AR07.

References

1. Van Laethem, K.; Schrooten, Y.; Dedecker, S.; van Heeswijck, L.; Deforche, K.; van Wijngaerden, E.; van Ranst, M.; Vandamme, A.M. A genotypic assay for the amplification and sequencing of gag and protease from diverse human immunodeficiency virus type 1 group M subtypes. *J. Virol. Methods* **2006**, *132*, 181–186.
2. Snoeck, J.; Riva, C.; Steegen, K.; Schrooten, Y.; Maes, B.; Vergne, L.; van Laethem, K.; Peeters, M.; Vandamme, A.M. Optimization of a genotypic assay applicable to all human immunodeficiency virus type 1 protease and reverse transcriptase subtypes. *J. Virol. Methods* **2005**, *128*, 47–53.
3. Van Laethem, K.; Schrooten, Y.; Covens, K.; Dekeersmaeker, N.; de Munter, P.; van Wijngaerden, E.; van Ranst, M.; Vandamme, A.M. A genotypic assay for the amplification and sequencing of integrase from diverse HIV-1 group M subtypes. *J. Virol. Methods* **2008**, *153*, 176–181.
4. Covens, K.; Dekeersmaeker, N.; Schrooten, Y.; Weber, J.; Schols, D.; Quiñones-Mateu, M.E.; Vandamme, A.M.; van Laethem, K. Novel recombinant virus assay for measuring susceptibility of human immunodeficiency virus type 1 group M subtypes to clinically approved drugs. *J. Clin. Microbiol.* **2009**, *47*, 2232–2242.
5. Liu, S.L.; Rodrigo, A.G.; Shankarappa, R.; Learn, G.H.; Hsu, L.; Davidov, O.; Zhao, L.P.; Mullins, J.I. HIV quasispecies and resampling. *Science* **1996**, *173*, 415–416.

© 2016 by the authors; licensee MDPI, Basel, Switzerland. This article is an open access article distributed under the terms and conditions of the Creative Commons by Attribution (CC-BY) license (http://creativecommons.org/licenses/by/4.0/).
